# An attachment‐based program for parents of youth with clinically significant mental health problems: Scaling up and drilling down to mechanisms of change

**DOI:** 10.1002/jcv2.12248

**Published:** 2024-05-17

**Authors:** Marlene M. Moretti, Sebastian P. Dys, Stephanie G. Craig, Carlos A. Sierra Hernandez, Natalie Goulter, Katherine O’Donnell, Dave S. Pasalich

**Affiliations:** ^1^ Department of Psychology Simon Fraser University Burnaby BC Canada; ^2^ Department of Psychology University of Guelph Guelph ON Canada; ^3^ College of Education Psychology and Social Work Flinders University SA Adelaide Australia; ^4^ School of Medicine and Psychology Australian National University Canberra ACT Australia

**Keywords:** adolescence, attachment, attachment‐based treatment, mechanisms of change, transdiagnostic factors, youth mental health

## Abstract

**Background:**

Given the prevalence and recent increases in youth mental health problems, there is a pressing need for interventions that target transdiagnostic protective factors that could be targeted as mechanisms of change in treatment. Such interventions are most likely to succeed in meeting population needs if they are scalable, sustainable, and effective. *Connect* is a manualized, 10‐session trauma‐informed and attachment‐based parent program that is structured, emotion‐focused and skills‐oriented. Developed for broad implementation by community mental health workers, *Connect* is designed to promote parent–child attachment security, a well‐established transdiagnostic protective factor for youth mental health.

**Methods:**

We examined whether parent–youth attachment anxiety and avoidance predicted reductions in internalizing and externalizing problems in a large one‐group clinical sample of youth (*N* = 527; ages 8–18 years) of parents (*N* = 690) who completed the *Connect* program in a longitudinal study with 6 time points (pre‐, mid‐, and post‐treatment; 6‐, 12‐ and 18‐month follow‐up).

**Results:**

Findings confirmed that parent and youth reports of attachment anxiety and avoidance, as well as internalizing and externalizing problems, significantly declined over the course of the intervention. Parent reported reductions in youth attachment anxiety, but not avoidance, predicted declining levels of youth internalizing problems. As well, parent reported reductions in youth attachment avoidance and anxiety predicted declining youth externalizing behavior. In contrast, youth reports of reductions in youth attachment anxiety, but not attachment avoidance, were associated with declines in youth externalizing problems.

**Conclusion:**

Our findings support the role of attachment as an important transdiagnostic mechanism of change in attachment‐based programs for parents of teens with clinically significant mental health problems.

## Graphical abstract

We longitudinally examined whether parent–youth attachment anxiety and avoidance predicted reductions in internalizing and externalizing problems in a large one‐group clinical sample of youth and their parents who completed *Connect*, an attachment‐based parenting program. Parent reports indicated that reductions in youth attachment anxiety predicted reductions in internalizing problems, whereas reductions in attachment anxiety and avoidance predicted reductions in externalizing problems.

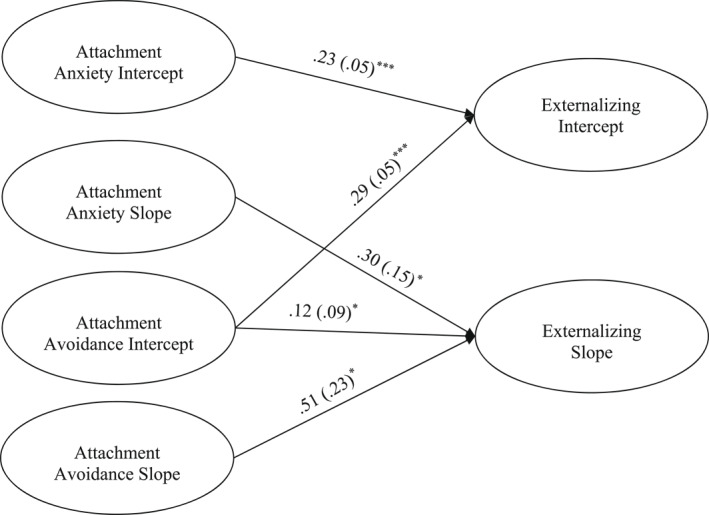

**Key points**

- According to youth and parents, *Connect* was effective at improving youth externalizing and internalizing problems, as well as youth–parent attachment.
- Brief, structured parent‐focused attachment‐based treatments may be effective transdiagnostic approaches to increasing youth attachment security and promoting youth mental health in the short and long term.
- Reductions in youth attachment anxiety and avoidance may be a pre‐requisite for changes in internalizing and externalizing problems among youth at high risk for behavior problems.
- More research is needed to examine additional mechanisms through which *Connect* improves youth mental health.

Rates of mental health disorders in youth have steadily grown over the past decade (e.g., Wiens et al., 2020), accompanied by rapid increases in the use of emergency, inpatient, and outpatient services (Kalb et al., 2019). Despite rising awareness of mental health problems among youth, the development, implementation, and accessibility of evidence‐based interventions tailored to the specific challenges of adolescents and their parents remain limited. Interventions that target transdiagnostic factors that mitigate risk may diminish a broad range of mental health problems among youth and are potentially more cost efficient than narrowly focused treatments (e.g., Weisz et al., 2019).

Insecure attachment is a transdiagnostic risk factor for mental health problems. Unlike attachment security—which involves a strong disposition to seek comfort and proximity with a caregiver when support is needed—attachment insecurity involves relying more heavily on secondary coping strategies to navigate stressful experiences (Ainsworth et al., 1978; Fearon et al., 2010). Attachment insecurity includes attachment anxiety, which involves a worry that others will not be responsive to oneself in times of need, as well as attachment avoidance, which involves not seeking proximity or contact with a caregiver when distressed (Ainsworth et al., 1978; Collins & Feeney, 2004). In a recent meta‐analysis of 40 samples including 2927 adults, Herstell et al. (2021) found that healthy controls were distinguished from those with major depression, bipolar disorder, and schizophrenia by a large effect size in anxious and avoidant attachment (*g* = 0.94 and 0.63, for anxious and avoidant attachment respectively). Likewise, insecure attachment in youth is associated with depression, anxiety, eating disorders, conduct and oppositional behaviors, substance misuse, and suicidality (e.g., Craig et al., 2020; Schindler, 2019).

Early theorizing posited that subtypes of attachment insecurity may be differentially related to internalizing versus externalizing symptomatology. Anxious attachment was believed to relate to internalizing problems because it inhibited the exploration of one's environment and thereby hampered the development of age‐appropriate affect regulation strategies (Cassidy & Berlin, 1994). Consequently, children would be ill equipped to handle experiences of fear or helplessness, making them vulnerable to depression and anxiety (Finnegan et al., 1996). Conversely, avoidant attachment was believed to relate to externalizing problems because it inhibited the development of emotional connectedness (e.g., empathy) and promoted a focus on satisfying one's own desires at a cost to others' rights and wellbeing, leading to exploitative and aggressive behaviors (Finnegan et al., 1996). Empirical findings have shown mixed support for these ideas, however. In terms of internalizing problems, meta‐analyses have found that adolescents and adults with anxious attachment representations experience more anxiety and depressive symptoms than those with secure attachment representations (*d* = 0.35 and 0.48, respectively) but the same was not true for those with avoidant attachment representations (*d* = −0.02 and 0.09, respectively; Dagan et al., 2018; Dagan et al., 2020). In terms of externalizing problems, far less research has examined these relations in adolescence. A meta‐analysis of child studies found a significant effect of avoidant attachment on externalizing problems and a non‐significant effect of anxious attachment, although the magnitude of the former was only slightly stronger (*d* = 0.12 and 0.11, respectively; Fearon et al., 2010). How these subtypes of attachment relate to mental health problems in adolescence remains an open question, especially given that meta‐analytic evidence that the link between attachment and externalizing problems increases with age (Fearon et al., 2010).

Attachment‐based treatments (ABTs) have been shown to be effective in reducing a range of behavioral and social‐emotional problems among infants and children (e.g., In Steele & Steele, 2017). A recent meta‐analytic review by Jugovac et al. (2022) found that randomized controlled trials of attachment‐ (and emotion‐) focused parenting interventions were associated with reductions of −0.30 and −0.17 standardized mean differences for internalizing and externalizing problems, respectively. Nevertheless, few ABTs have been developed for youth with clinically significant mental health problems. *Connect* is an innovative manualized, 10‐session, trauma‐informed and attachment‐based parent program for caregivers of youth (ages 8–18 years) with behavioral and mental health challenges. This parent group intervention combines emotion‐focused exercises, role‐plays, and reflective discussions (Fonagy & Luyten, 2018) to promote and parenting skill development. Parents practice “stepping back” to develop awareness of their distressing thoughts and feelings and those of their child. They also practice “stepping forward” to reflect on and be curious about attachment needs expressed through their child's behavior. Facilitators work to promote parental self‐compassion, self‐regulation, and the use of empathy to support emotion regulation in their relationship with their child. Rather than tell parents how to respond to challenges with their teen, parents reflect together to envision and practice various ways that they may be able “leave the door open” in their relationship with their child by responding with warmth and sensitivity that promotes safe haven and secure base while setting clear limits and expectations. Thus, *Connect* differs from traditional social learning approaches by shifting the primary program goals from behavior management and problem solving to promoting reflective capacity, emotion regulation, sensitive care and building security within the parent–adolescent relationship.

In a series of quasi‐experimental and randomized clinical trials (RCTs), *Connect* has been shown to reduce parent depressive symptoms, caregiver stress, parent–teen conflict, and reduce insecure attachment and mental health problems among teens at risk (Moretti & Obsuth, 2009; Stattin et al., 2015); with retention or deepening of treatment effects from post‐treatment to 2‐year follow‐up period (Högström et al., 2017). *Connect* is also flexible and can be adapted to cultural, gender, and other diversities. In an RCT using a culturally adapted version of *Connect* with 120 Somali‐born parents forcibly dislocated and living in Sweden, significant reductions were found in internalizing and externalizing problems in youth of parents in the treatment versus wait‐list condition (Osman et al., 2017), and these outcomes were retained at three years post‐treatment (Osman et al., 2021). A small preliminary RCT using an adapted version of *Connect* for kinship parents also found that those who completed the program reported significantly greater reductions in youth mental health problems, caregiver strain, and fewer unplanned placements, compared to those who received support‐as‐usual (Pasalich et al., 2021). In terms of subtypes of attachment insecurity, some evidence has been found for differential effects over the course of *Connect*. A nonrandomized trial found that reductions in parents' reports of youth attachment anxiety were associated with decreases in youth internalizing symptoms, whereas reductions in youth attachment avoidance were associated with decreases in youth externalizing symptoms (Moretti et al., 2015). Furthermore, two RCTs of *Connect* replicated these findings, with decreases in anxious, but not avoidant, attachment longitudinally predicting decreases in internalizing problems and decreases in avoidant, but not anxious, attachment predicting decreases in externalizing problems (Barone et al., 2021).

Together this research suggests *Connect* effectively mitigates a broad range of mental health problems in youth and improves parent functioning, but little is known about the mechanisms that underlie these treatment effects. From a theoretical perspective, ABTs are presumed to increase parent–child attachment security which, in turn, is believed to promote mental health and wellbeing. Our prior research provides preliminary support of this view: using the parenting representation interview (Scharf et al., 2015), we found that parental states of mind reflected increased attachment security in the parent–teen relationship over treatment, and this was associated with decreases in child problem behavior (Moretti et al., 2012). Likewise, parents who completed *Connect* have reported significant pre‐to‐post treatment reductions in youth attachment anxiety and avoidance which are related to reductions in youth internalizing and externalizing (Moretti et al., 2015). While promising, these studies have not examined the cross‐time temporal associations between reductions in youth attachment anxiety and avoidance, and youth outcomes, nor have they examined these changes across longer periods of follow‐up. The objective of the current study was to examine changes in youth attachment and mental health functioning among youth of parents enrolled in the *Connect* program across six time‐points, from pre‐treatment to 18‐month follow‐up. Past intervention studies are also limited in their reliance parent reports even though youth reports may produce different findings. For example, in an evaluation of parent programs, Jalling et al. (2016) found while parent reports showed significant reductions in youth antisocial behavior in the treatment versus control condition while youth reported a threefold increase in substance use in both conditions. The current study examines patterns of change in both parent and youth reports of attachment and youth mental health problems to gain a fuller understanding of attachment as a mechanism of change in treatment.

## CURRENT STUDY

Using a longitudinal one‐group design, we examined changes in parent–child attachment anxiety and avoidance in relation to changes in internalizing and externalizing problems among youth of parents who completed the *Connect* program. Parallel process models were used to examine these associations using parent and youth reports over 6 time points (pre‐, mid‐, and post‐treatment; 6‐, 12‐ and 18‐month follow‐up) in a sample of 690 families referred for services due to their child's social, emotional, and behavioral problems. Based on prior research, we expected to find significant reductions in youth and parent reports of attachment anxiety and avoidance, as well as youth internalizing and externalizing problems from pre‐treatment through follow‐up. We further predicted that reductions in parent and youth reports of attachment anxiety and avoidance would predict declines in youth internalizing and externalizing, respectively, across time.

## METHOD

### Participants

Participants were 690 birth parents of children 7.5 years and older (age 24–65, *M*_age_ = 42.86 years, *SD* = 6.88; 86.4% mothers; ethnicity: 74.9% white; 9.6% Indigenous; 6.4% Asian; 4.5% other; 4.6% not disclosed). Family income fell below poverty line for almost half of the sample (47.4%); 23% of parents reported partial or full high‐school parent education; 63.3% partial or full college/university; 3.8% post‐graduate; 7.0% missing. Youth (*N* = 527) were 8–19 years of age (*M*_age_ = 14.00 years, *SD* = 2.31; 56.4% female; ethnicity: 59.8% white; 10.4% Indigenous; 4.6% Asian; 8.0% other; 17.3% not disclosed). The contextualize these youths' mental health problems, we examined rates of clinical, subclinical, and elevated internalizing and externalizing psychopathology at pre‐intervention via parent ratings on the Brief Child and Family Phone Interview (BCFPI; see *Measures*) as indicated by *T*‐scores of greater than 70, 65, and 60, respectively. For internalizing problems, 42.0% met the clinical threshold, with another 11.6% scored in the subclinical range and 16.6% fell in the elevated range. For externalizing problems, 55.9% of the sample met the clinical threshold, with another 14.2% scored in the clinical range and 11.2% fell in the elevated range. Only 3.5% of the sample did not meet at least the elevated threshold of either internalizing or externalizing problems.

### Procedure

Parents referred from community mental health services for youth mental health problems were invited to enroll in *Connect* program in urban and rural communities in Canada. Exclusion criteria were youth psychosis, schizophrenia, or acute mental health condition requiring urgent hospitalization, or low intellectual functioning (IQ < 70). Parents provided consent for themselves and their child for participation; youth also provided assent for participation. We used a one‐group design, meaning no parents were assigned to control conditions. Parent and youth questionnaire measures were completed prior to *Connect*, at mid‐treatment (session five), post‐treatment and at 6‐, 12‐ and 18‐month follow‐up. Caregivers and youth each received a $25 honorarium at each time point. All study protocols and procedures were approved by the university research ethics board.

### Connect parent program

*Connect* sessions require two facilitators to deliver role‐plays, reflection exercises and to manage group dynamics. Facilitators completed a 3‐day *Connect* training workshop and received weekly supervision based on reviews of their videotaped sessions to achieve certification. The program begins with a pre‐treatment engagement meeting with each parent designed to build trust, promote motivation, and resolve treatment barriers, followed by a welcome meeting for the group, nine 1.5‐h program sessions, and a final meeting in which parents reflect on and provide feedback about their experiences in the program.

### Measures

#### Youth attachment

Parents and youth completed the brief 16‐item Adolescent Attachment Anxiety and Avoidance Inventory (AAAAI; Moretti & Obsuth, 2009) that measures attachment anxiety (e.g., “My child worries about being abandoned by me”) and attachment avoidance (e.g., “My child tries to avoid getting too close to me”). The AAAAI has established a robust factor structure and reliability (Barone et al., 2021; Moretti et al., 2015; Moretti & Obsuth, 2009; Pasalich et al., 2021). This measure takes a dimensional, rather than categorical, approach to attachment, which is commonly regarded as the preferred approach for assessing attachment (Fraley et al., 2015; Raby et al., 2020). Items are rated on 1 (*strongly disagree*) to 7 (*strongly agree*) point scale, and average item subscale scores are generated. Cronbach's alpha for parent and youth reports were strong over time (Parents: anxiety: α = 0.83–0.87; avoidance: α = 0.91–92; Youth: anxiety: α = 0.83–0.90; avoidance: α = 0.90–0.92, respectively).

#### Youth internalizing and externalizing problems

The Brief Child and Family Phone Interview (BCFPI; Boyle et al., 2009; Cunningham et al., 2009) a broad spectrum measure of youth psychopathology, shows good agreement with interview based diagnoses (Andersson et al., 2018) and generates six subscales, approximating diagnostic criteria for separation anxiety disorder, generalized anxiety disorder, major depressive disorder, attention‐deficit/hyperactivity disorder, oppositional‐defiant disorder, and conduct disorder, which in turn produce scale scores for internalizing and externalizing symptoms. Cronbach's alpha for parent‐ and youth‐reported internalizing and externalizing scales were strong over time (parent report: α = 0.88–0.90; α = 0.87–90 respectively; youth report: α = 0.92–0.93 and α = 0.86–0.87 respectively).

### Attrition and missing data

Parents who had children participate in the study did not differ at pre‐treatment on demographic or study variables from those who did not. To account for missing data at the item level, scales were computed when at least 75% of items within each scale were present (Newman, 2014) at each point in time and examined for whether cases with or without missing data differed at pre‐treatment on study variables. Parent data missingness at the 6‐ and 18‐month follow‐ups was related to lower parent education and income. Youth data missingness did not differ on demographic variables at pre‐treatment; however, missingness at mid‐treatment and 18‐month follow‐up was related to higher pre‐treatment internalizing problems and attachment avoidance. All models in the final analyses were estimated using full‐information maximum likelihood (FIML).

Parents completion of questionnaire packages was 97.7% at pre‐treatment; 80.9% at mid‐treatment; 74.6% at post‐treatment; 57.2% at 6‐month follow‐up, 50.0% at 12‐month follow‐up, and 60.7% at 18‐month follow‐up. Youth completion was 93.2% at pre‐treatment; 79.9% mid‐treatment; 74.7% at post‐treatment; 55.3% at 6‐month follow‐up; 49.4% at 12‐month follow‐up; 58.8% at 18‐month follow up.

### Statistical analysis

Analyses were completed using *Mplus* Version 8.2 (Muthén & Muthén, 2019). Latent growth curve modeling (LGCM) for youth attachment anxiety and avoidance as well as internalizing and externalizing symptoms were used to assess the initial levels (i.e., intercept) and trajectory of change (i.e., slope) over‐*Connect* and follow‐up. Parallel process models were fitted to allow us to model the interrelations between the intercept and slope of both attachment anxiety and avoidance with the intercept and slope of internalizing or externalizing problems. Variability in the duration of time elapsed between assessment periods was addressed by fixing the intercept factor loadings to 1 but allowing the linear slope factor loadings to vary from pre‐treatment to 18‐month follow‐up. Due to the large variances associated with *T*‐scores, all *T*‐scores were transformed by a division of 10 for all LGC analysis; thus, means reported for psychopathology *T*‐scores are 10 times smaller than observed. In all LGC models, we controlled for youth age and gender, parent gender, and group attendance. To address potential issues of directionality, we conducted supplemental post‐hoc analyses using the random intercepts cross‐lagged panel modeling (RI‐CLPM; Hamaker et al., 2015). Doing so offers more insight into the directionality between attachment and mental health problems, especially when LGC models find the slopes of two variables to be related over the same period. The RI‐CLPM can offer more fine‐grained insights into how changes from individual assessment points predict changes in subsequent timepoints. For instance, finding that changes in attachment from T2 (mid‐intervention) onward predict subsequent longitudinal changes in internalizing or externalizing problems, would provide evidence of directionality that is consistent with our expectation that attachment is a mechanism through which *Connect* improves mental health outcomes. All models were evaluated according to the most commonly used critical values for the fit indices (Hu & Bentler, 1999). The data that support all the findings of this study are available from the first author upon reasonable request.

## RESULTS

In terms of group attendance, 84.7% attended 6 of the 9 program sessions (considered to be fully completed) of which 50.4% attended all sessions. Means and standard deviations for all variables of interest can be found in Table 1.

**TABLE 1:** Means and standard deviations for control variables and variables of interest at each time point.

	Parent report	Youth report
Variable	*M* (*SD*)	*M* (*SD*)
Age	42.87 (6.97)	14.00 (2.31)
Number of sessions	7.75 (2.41)	
Externalizing		
Time 1	71.79 (13.00)	61.53 (12.48)
Time 2	65.20 (11.73)	56.67 (12.20)
Time 3	62.73 (11.94)	55.07 (12.20)
Time 4	65.42 (12.71)	58.01 (12.12)
Time 5	65.13 (13.13)	57.74 (12.80)
Time 6	65.28 (12.96)	57.40 (12.01)
Internalizing		
Time 1	67.89 (14.33)	58.56 (12.53)
Time 2	59.99 (13.12)	53.38 (12.95)
Time 3	57.51 (12.94)	52.42 (13.19)
Time 4	62.10 (13.25)	54.71 (13.11)
Time 5	61.36 (13.72)	55.34 (12.60)
Time 6	61.04 (13.4)	54.69 (12.52)
Attachment anxiety		
Time 1	3.28 (1.28)	2.70 (1.27)
Time 2	3.12 (1.20)	2.51 (1.32)
Time 3	3.02 (1.21)	2.39 (1.30)
Time 4	2.97 (1.23)	2.44 (1.27)
Time 5	2.93 (1.22)	2.38 (1.28)
Time 6	2.88 (1.23)	2.34 (1.28)
Attachment avoidance		
Time 1	3.24 (1.31)	3.77 (1.50)
Time 2	3.09 (1.28)	3.78 (1.52)
Time 3	2.98 (1.21)	3.63 (1.50)
Time 4	2.92 (1.23)	3.55 (1.48)
Time 5	2.94 (1.29)	3.44 (1.53)
Time 6	2.92 (1.25)	3.36 (1.39)

### Parent models

Unconditional LGC models for parent reported symptoms and attachment can be found in Supporting Information S1. All LCG models controlled for youth age and gender, parent gender, and group attendance. The unconditional models all fit the data well and found significant decreases in internalizing and externalizing symptoms and attachment avoidance and anxiety from pre‐treatment to 18 months follow up.

**FIGURE 1:**
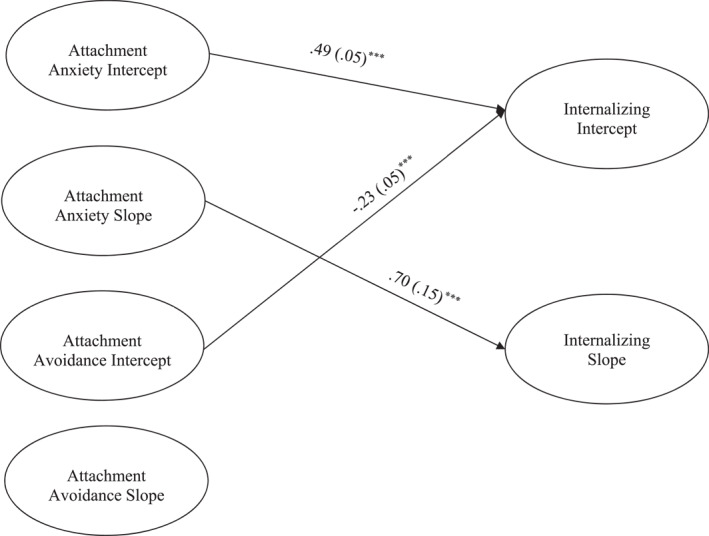
Parent Report Latent Growth Curve model depicting attachment predicting initial levels and changes in internalizing symptoms. ***p ≤ 0.001. Only significant paths are shown. Parameter estimates represent standardized coefficients with standard errors in brackets. Not shown: model controlling for attendance, parent and youth gender, and youth age.

Next, we conducted parallel process models where we tested whether attachment anxiety and avoidance changes in and externalizing problems across the intervention and to 18‐month follow‐up. Only significant pathways are discussed. In the internalizing model, due to a non‐significant negative residual variance, the residual variance of the internalizing slope was set to 0. The model for attachment predicting internalizing problems fit the data well (χ^2^ (181) = 410.53, *p* ≤ 0.001, *CFI *= 0.96, *RMSEA* = 0.04, 90% CI [0.04, 0.05]; see Figure 1). In terms of covariates, older children scored higher on starting levels of internalizing problems (β = 0.14, *p* ≤ 0.001) and higher attendance was related to a steeper decline in internalizing problems (β = −0.45, *p* ≤ 0.001).

The intercept for internalizing was predicted by higher initial levels of attachment anxiety (β = 0.49, *p* ≤ 0.001) and lower initials levels of avoidance (β = −0.23, *p* ≤ 0.001). The slope for internalizing was predicted by the slope for attachment anxiety (β = 0.70*, p* ≤ 0.001), indicating steeper declines of attachment anxiety predicted steeper declines in internalizing problems. The intercept and slope for internalizing was not associated with attachment avoidance.

**FIGURE 2:**
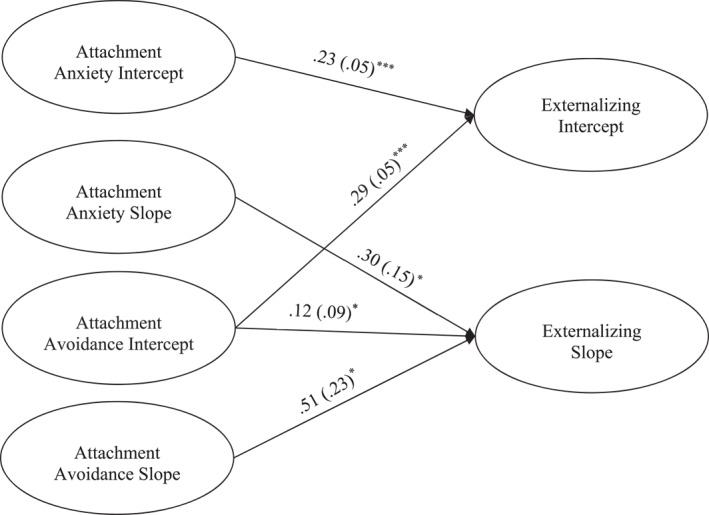
Parent report Latent Growth Curve model depicting attachment predicting initial levels and changes in externalizing symptoms. *p < 0.05, ***p ≤ 0.001. Only significant paths are shown. Parameter estimates represent standardized coefficients with standard errors in brackets. Not shown: model controlling for attendance, parent and youth gender, and youth age.

For the externalizing model, the model fit the data well (χ^*2*^ (181) = 517.56, *p* ≤ 0.001, *CFI* = 0.94, *RMSEA* = 0.05, 90% CI [0.05, 0.06]; see Figure 2). The intercept for externalizing was associated with higher levels of both attachment anxiety intercept (β = 0.22, *p* ≤ 0.001) and avoidance intercept (β = 0.30, *p* ≤ 0.001). The slope for externalizing was associated with the intercept for avoidance (β = 0.21, *p* = 0.02), indicating that lower pre‐treatment avoidance was associated with a steeper decrease in externalizing problems. The slopes of attachment avoidance (β = 0.51, *p* = 0.02) and anxiety (β = 0.30, *p* = 0.05) were associated with externalizing, indicating steeper declines in both were associated with steeper decreases in externalizing behavior.

As a further test of our prediction that changes in youth attachment led to changes in youth mental health functioning, we tested the alternate parallel process models in which changes in internalizing and externalizing problems predicted attachment anxiety and avoidance. The model testing whether internalizing problems predicted attachment anxiety and avoidance was acceptable (χ^2^ (184) = 441.57, *p* < 0.001, *CFI* = 0.95, *RMSEA* = 0.05, 90% CI [0.04, 0.05]), however the slope for internalizing was not associated with the attachment anxiety slope (β = 0.03, *p* = 0.72) or avoidance (β = −0.11, *p* = 0.12). The model for externalizing problems predicting attachment anxiety and avoidance was also acceptable (χ^2^ (181) = 566.12, *p* < 0.001, *CFI* = 0.93, *RMSEA* = 0.06, 90% CI [0.05, 0.06]). While the slope of externalizing did not predict the attachment anxiety slope (β = 0.56, *p* = 0.18), it did predict the avoidance slope (β = 0.72, *p* = 0.002).

### Youth models

Unconditional LGC models for youth reported symptoms and attachment can also be found in Supporting Information S1. All models controlled for youth age and gender, parent gender, and group attendance. The unconditional models fit the data well. In the internalizing model, due to a non‐significant negative residual variance, the residual variance of the internalizing slope was set to 0. Internalizing and externalizing symptoms significantly decreased, as did attachment anxiety and avoidance, according to youth reports.

**FIGURE 3:**
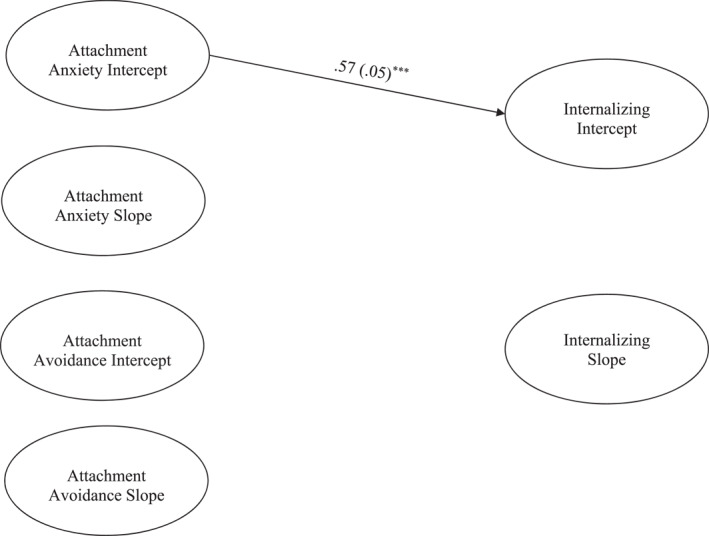
Youth Report Latent Growth Curve model depicting attachment predicting initial levels and changes in internalizing symptoms. ***p ≤ 0.001. Only significant paths are shown. Parameter estimates represent standardized coefficients with standard errors in brackets. Not shown: model controlling for attendance, parent and youth gender, and youth age.

Next, we conducted parallel process models to test whether attachment anxiety and avoidance predicted changes in internalizing and externalizing problems across the intervention and to 18‐month follow‐up. Only significant pathways are discussed. The parallel process model for attachment predicting internalizing problems fit the data well (χ^2^ (173) = 443.46, *p* ≤ 0.001, *CFI* = 0.92, *RMSEA* = 0.05, 90% CI [0.05, 0.06]; see Figure 3). The internalizing intercept was predicted by the attachment anxiety intercept (β = 0.57, *p* < 0.001). The internalizing slope was not predicted by the attachment anxiety or avoidance intercept or slope.

**FIGURE 4:**
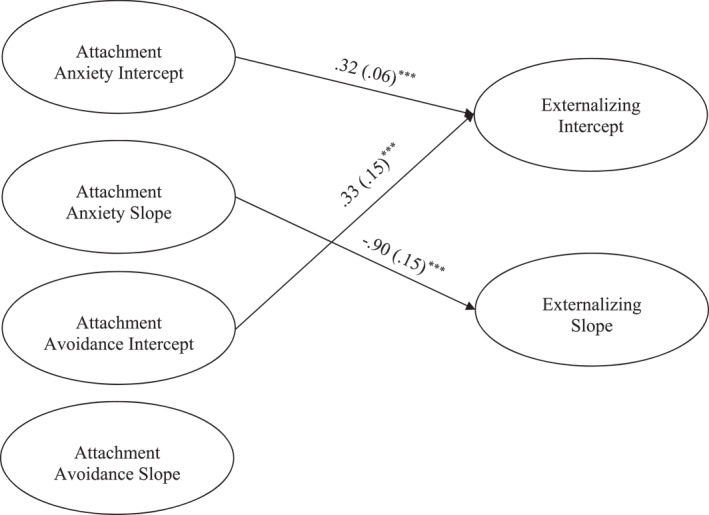
Youth Report Latent Growth Curve model depicting attachment predicting initial levels and changes in externalizing symptoms. **p < 0.01, ***p ≤ 0.001. Only significant paths are shown. Parameter estimates represent standardized coefficients with standard errors in brackets. Not shown: model controlling for attendance, parent and youth gender, and youth age.

The model for attachment predicting externalizing problems fit the data well (χ^2^ (172) = 427.04, *p* ≤ 0.001, *CFI *= 0.93, *RMSEA* = 0.05, 90% CI [0.05, 0.06]; See Figure 4). The attachment anxiety intercept (β = 0.32, *p* < 0.001) and avoidance intercept (β = 0.33, *p* = 0.02) significantly predicted the externalizing intercept, such that higher levels of pre‐treatment attachment anxiety and avoidance were associated with higher initial levels of externalizing. The externalizing slope was only predicted by the attachment anxiety slope (β = −0.90, *p* < 0.001), such that steeper decreases in attachment anxiety were associated with slower decreases in externalizing. Surprisingly, attachment avoidance was not associated with the externalizing slope.

To further test whether changes in youth reported attachment anxiety and avoidance temporally preceded changes in internalizing and externalizing problems, we examined alternate parallel process models in which internalizing and externalizing problems predicted attachment anxiety and avoidance. The model in which internalizing predicted attachment anxiety and avoidance was acceptable (χ^2^ (173) = 609.16, *p* ≤ 0.001, *CFI* = 0.91, *RMSEA* = 0.06, 90% CI [0.06, 0.07]). However, the slope of internalizing did not significantly predict the attachment anxiety slope (β = 0.67, *p* = 0.07) or the avoidance slope (β = 0.36, *p* = 0.12). Likewise, the model in which externalizing predicted attachment anxiety and avoidance was also acceptable (χ^2^ (175) = 408.92, *p* ≤ 0.001, *CFI* = 0.94, *RMSEA* = 0.05, 90% CI [0.04, 0.06]). However, the slope of externalizing did not predict the attachment anxiety slope (β = 0.20, *p* = 0.63) or the avoidance slope (β = 0.43, *p* = 0.62).

To further test the directional nature of attachment and mental health problems, we ran four random‐intercept cross‐lagged panel models (RI‐CLPM), which allow us to examine, at each timepoint, how within‐person variability in attachment predicts subsequent within‐person variability in mental health problems. The findings are detailed in Supporting Information S1. In short, our models generally supported the idea that changes in attachment preceded changes in mental health problems. Parent‐reported models found changes in attachment anxiety to predict changes in internalizing problems as well as changes in attachment avoidance predicting changes in externalizing problems, but not the inverse. The youth‐reported externalizing model found attachment avoidance to predict externalizing problems, which later predicted attachment anxiety. The youth‐reported internalizing model, however, found that internalizing problems predicted attachment avoidance, but not the inverse.

## DISCUSSION

We evaluated the role of attachment as mechanism across six time‐points during and following the delivery of an innovative ABT delivered to a large sample of parents of youth with clinically significant mental health problems. Strong uptake and completion of mental health services is critical to optimal service utilization. Despite the clinical nature of our sample, 85% of parents completed the *Connect* program, which is promising compared to the estimated 60% retention rate (40% drop out) for parent training programs that target disruptive child behaviors (see Koerting et al., 2013). Our retention may be due in part to our invitational meeting with parents prior to the start of *Connect*, which uses motivational interviewing and collaborative problem solving to address barriers to program attendance. The relationally focused, strength based and autonomy promoting focus of the program also aims to boost parents' sense of inclusion and engagement, and to minimize feelings of parental shame, creating trust with and among parents. Our findings add to prior research by showing reductions in youth attachment anxiety and avoidance, and internalizing and externalizing problems, irrespective of whether we examined parent or youth reports. Overall, the results also lend some support to the role of attachment as a change mechanism that drives reductions in youth mental health problems, although this support was mostly found in parent, but not youth, reports.

In terms of attachment anxiety, we found that steeper parent‐reported declines predicted falling levels of internalizing problems from pre‐treatment to 18‐month follow up. These findings mirror prior research (Barone et al., 2021), suggesting a unique relation between youth attachment anxiety and symptoms of depression and anxiety. For youth reports, we found relations between the intercepts of attachment anxiety and internalizing problems, but not their slopes. A possible explanation centers around systematic informant discrepancies: when reporting on their youth–parent relationship, youth with higher levels of depressive symptoms show greater deviations from their parents' ratings than youth with lower levels of depressive symptoms (De Los Reyes et al., 2008). Therefore, it is possible that youths' depressive symptoms, predictably, were related to their internalizing scores, but also, less predictably, distorted perceptions of their attachment with their parents. Additionally, it could be that this discrepancy reflected differences between the settings in which parents and youth observe youth; for example, youth might base their ratings on their behavior at home, at school, and during unsupervised leisure time with friends, whereas parents might only consider their youths' behavior at home. Therefore, youths' ratings may reflect their own behavior in a more general manner, whereas parents' ratings of youths' behavior might be more situation specific. It is also possible that other mechanisms affected by *Connect* are more impactful on youths' perceptions of their mental health than their parent–youth attachment. Alternatively, these null findings may also reflect false‐negative (Type II) errors.

Surprisingly, we also found that decreases in youth‐reported attachment anxiety were inversely related to youth‐reported externalizing problems. For some youth, a decrease in attachment anxiety may make them more comfortable and secure to explore some externalizing‐related behaviors, especially those are that more common among adolescents, as they feel less threatened by the repercussions of their actions on their relationship with their caregiver, but this idea requires further investigation. Future studies may opt to examine these relations using person‐centered approaches to examine whether these group level findings have masked subgroup differences in how changes in attachment anxiety related to changes in internalizing and externalizing problems over the course of *Connect*.

The role of youth attachment avoidance in treatment change was less clear. Although we found that parent‐reported reductions in avoidance (but also attachment anxiety), predicted declining externalizing behavior, we did not find similar patterns in youth‐reported data. At the same time, our supplemental analyses pointed to predictive relations of attachment avoidance on externalizing problems, across both parent and youth reports. Surprisingly, our primary analyses found that lower rather than higher parent reported youth avoidance at pre‐treatment predicted steeper declines in problem behavior. It is possible that lower pre‐treatment levels of attachment avoidance were associated with higher levels of youths' openness to parents' intervention‐encouraged behaviors and fostered more connectedness to parents, youth disclosure, and parental monitoring. These findings around attachment avoidance were mostly inconsistent with previous findings (e.g., Barone et al., 2021; Moretti et al., 2015) and suggest that further research is needed to examine whether changes in attachment avoidance play a role in improving youth externalizing behavior, and if so, to what extent.

More generally for youth reports, the lack of hypothesized associations between changes in youth attachment and mental health outcomes was also surprising, especially since youth also reported improvements in attachment and mental health variables. In some cases, these divergences between findings with parent versus youth reports might reflect valid differences between youth and parent perceptions of the same constructs, based on the differences in which setting(s) youth behavior is observed in. It is possible that the mechanisms through which *Connect* exerts positive influences on youth mental health across settings—which are more likely reflected in youths' reports of their own behavioral problems—were not included in our analyses. Therefore, several factors that have been linked to youth mental health, such as youth emotion regulation (Compas et al., 2017), parental stress (Barroso et al., 2018), and harsh parenting (Pinquart, 2021), may warrant examination as potential mechanisms of change related to youth reports. Also, despite our youth sample size, another possibility is that the null findings for youth reports are false‐negative errors resulting from random sampling error, an insufficient sample size given the true effect size, or a combination thereof.

Nevertheless, these results generally suggest that reductions in youth attachment insecurity may be critical in laying the groundwork for further and lasting change. This is especially important in promoting mental health during adolescence, a time when youth are likely to have conflict with their parents and to question the value of their guidance and values. Reducing youth attachment anxiety may promote greater trust in parents as sensitive, trustworthy, and reliable, opening the door for better youth–parent communication and autonomy support. Still, more multi‐informant or observational work with attachment‐based programs with youth are needed to elucidate the role of attachment insecurity, and particularly avoidant attachment. The lack of relations between attachment avoidance and internalizing, however, was consistent with previous findings (e.g., Barone et al., 2021; Moretti et al., 2015).

Taken together, our results are consistent with the view that changes in youth attachment promote changes in youth mental health problems, but the reverse could be argued—i.e., that reductions in youth mental health problems promote attachment security. Yet, when we tested alternative models wherein youth internalizing and externalizing problems predicted changes in youth attachment anxiety and avoidance, little support emerged. We did, however, find in an alternative model that decreases in parents' reports of externalizing problems predicted reductions in youth attachment avoidance. The findings of this sensitivity test may warrant reducing some confidence in attachment avoidance as a mechanism of change (see Pizer, 2016). Still, our model predicting externalizing problems from attachment fit the data better than the alternative and showed confluence with our supplemental RI‐CLPM analyses for both informants, suggesting that changes in attachment avoidance preceded changes in externalizing problems. Taken together, the balance of these findings suggests that changes in attachment may be a pre‐requisite for therapeutic improvement in mental health among adolescents with clinical levels of mental health problems. While it is likely that a cascade of complex transactions between changes in youth attachment and mental health problems occurred over the course of intervention, our results suggest that attachment might hold foremost in this process.

Although promising, there are several limitations to keep in mind when interpreting our findings. First, this study is not an RCT and thus we cannot be certain that the results we observed were due to the completion of the *Connect* program, or that other programs may not achieve comparable results. Second, although we assessed both youth and parent indicators of attachment and youth mental health, these were questionnaire measures. While Herstell et al. (2021) make a strong case for questionnaire measures of risk factors, further studies using interview and observational measures are needed. Furthermore, our models involved the same informants to assess youth attachment and mental health. We chose this route given questions about the meaning of systematic informant discrepancies of youth mental health (De Los Reyes et al., 2023), and considering findings suggesting that shared method variance is accounted for in longitudinal estimates in models where between‐individual differences are accounted for (see Orth et al., 2022). Still, it is possible some shared informant variance has remained in our models. Future approaches may benefit from emerging models of multi‐informant analyses which involve separating common and setting‐specific variance (see De Los Reyes et al., 2023). Third, even though our results suggest that attachment may be a critical mechanism of change in ABTs such as the *Connect* program, like most parenting interventions, more than one mechanism is likely to drive treatment outcomes. Further research should adopt a stepwise approach to examining the impact of attachment alongside associated mechanisms such as parental depression, parenting stress, emotion regulation, parenting behaviors, and parents' own attachments to promote positive treatment outcomes and employ person‐centered approaches to better understand which mechanisms are predictive for whom. Doing so can provide more robust tests of the strength and specificity of mechanisms, as well as better evaluate our claims about attachment as a central mechanism of change in *Connect* (see Kazdin, 2007). Such endeavors, however, may require much larger sample sizes or the collection of intensive longitudinal data (e.g., ecological momentary assessments) when examining potential mediators alongside time varying predictors in a latent growth curve modeling framework to obtain sufficient statistical power (see Sullivan et al., 2021; Thoemmes et al., 2010).

## CONCLUSIONS

The current results add to the growing findings that ABTs may offer a viable alternative to addressing clinical levels of behavioral and other problems among teens, given the apparent limited effectiveness of social‐learning approaches (e.g., Hawes & Dadds, 2005). Indeed, in prior research we have found that youth with the highest levels of conduct problems and callous‐unemotional (CU) features responded the fastest and the most significantly from parents' completion of the *Connect* program (Pasalich et al., 2022). Based on our current findings, it may be that reducing attachment anxiety and avoidance, particularly early in treatment, is paramount to achieving significant and lasting reductions in conduct problems and CU traits. Programs that promote sensitive and positively focused parenting strategies are possibly suited for this population (e.g., Kimonis et al., 2019).

## AUTHOR CONTRIBUTION

**Marlene M. Moretti**: Conceptualization; data curation; funding acquisition; investigation; methodology; project administration; resources; supervision; writing – original draft; writing – review & editing. **Sebastian P. Dys**: Formal analysis; writing – review & editing. **Stephanie G. Craig**: Data curation; formal analysis; methodology; writing – review & editing. **Carlos A. Sierra Hernandez**: Data curation; formal analysis. **Natalie Goulter**: Data curation; formal analysis; supervision; writing – review & editing. **Katherine O’Donnell**: Data curation. **Dave S. Pasalich**: Formal analysis; investigation; methodology; supervision.

## CONFLICT OF INTEREST STATEMENT

Marlene M. Moretti occasionally receives remuneration for speaking and training engagements related to the *Connect* program. All other authors have declared they have no competing or potential conflicts of interest.

## ETHICAL CONSIDERATIONS

The study received ethics approval from the Simon Fraser University Office of Research Ethics [protocol #2011s0284].

## Supporting information

Supporting Information S1

## Data Availability

The data that support the findings of this study are available upon reasonable request from the first author.
