# Supplementary material for: An attachment‐based program for parents of youth with clinically significant mental health problems: Scaling up and drilling down to mechanisms of change
Source: JCPP Adv. 2024 May 17;5(1):e12248. doi: 10.1002/jcv2.12248 (PMC11889652; doi:10.1002/jcv2.12248)
Supplement: Supplementary file 1 — Supporting Information S1 [file JCV2-5-e12248-s001.docx]

**Supporting Information A**

**Parent LGC Models**

To ensure the means and intercepts for each variable were significant, unconditional models were independently specified for internalizing and externalizing problems. Parent-reports of internalizing problems fit the data well (χ^2^(8) = 8.30, *p* = .40, CFI = 1.00, RMSEA = .01 [.00, .04]). The intercept (*M* = 6.78, *p* < .001) and slope (*M* = -.60, *p* < .001) were both significant, indicating a significant decrease of internalizing problems from pre-treatment to 18-months. The internalizing intercept was associated with the slope (β = -.80, *p* < .001), indicating higher pre-treatment internalizing problems was associated with a steeper decline over treatment. Likewise, parent-report of externalizing problems fit the data well (χ^2^(7) = 9.94, *p* = .27, CFI = .99, RMSEA = .01 [.00, .05]). The intercept (*M* = 7.16, *p* < .001) and slope (*M* = -.63, *p* < .001) were both significant, indicating a significant decrease in externalizing problems from pre-treatment to 18-month. The externalizing intercept was associated with the slope (β = -.61, *p* < .001), indicating higher pre-treatment externalizing problems was associated with a steeper decline. The unconditional model for attachment anxiety also fit the data well (χ^2^(11) = 20.03, p = .05, CFI = .99, RMSEA = .04 [.01, .06]). The means for the anxiety intercept (*M* = 3.28, *p* < .001) and slope (*M* = -.33, *p* < .001) demonstrated a significant intercept and a decreasing slope across the treatment. The intercept of attachment anxiety was related to the slope (β = -.39, *p* < .001), such that higher pretreatment attachment anxiety was related to a steeper decline over treatment. Similarly, the model for attachment avoidance fit the data well (χ^2^(11) = 21.94, *p* = .03, CFI = .99, RMSEA = .04 [.01, .06]). The intercept (*M* = 3.08, *p* < .001) and slope (*M* = -.10, *p* = .04), indicated a significant mean and decreasing slope across the time points. The intercept of avoidance was related to the slope (β = -.37, *p* < .001), such that higher pre-treatment attachment avoidance was related to a steeper decline.

**Youth LGC Models**

Due to negative and nonsignificant residual variances, the variance of both internalizing and externalizing problems were set to 0 in the unconditional model. The model fit for internalizing was acceptable (χ^2^(11) = 26.68, *p* = .001, CFI = .98, RMSEA = .05[.03, .07]). The intercept (*M* = 5.87, *p* < .001) and slope (*M* = -.36, *p* < .001) were both significant, indicating a significant decrease of internalizing from pre-treatment to 18-months follow-up. The model fit for externalizing was also acceptable (Χ2(11) = 25.38, p = .001, CFI = .99, RMSEA = .05 [.02, .08]). The intercept (*M* = 6.16, *p* < .001) and slope (*M* = - .36, *p* < .001) were both significant, indicating a significant decrease of externalizing from pre-treatment to 18-months follow-up. Attachment anxiety fit the unconditional model well (χ^2^(11) = 16.19, *p* = .13, CFI = .99, RMSEA = .03 [.00, .06]). The intercept (*M* = 2.48, *p* < .001) and slope (*M* = -.07, *p* = .04) were both significant indicating a significant decrease of anxiety from pre-treatment to 18-months follow-up. Attachment avoidance also fit the data well (χ^2^(12) = 19.17, p = .08, CFI = .99, RMSEA = .03 [.00, .06]). The avoidance intercept (*M* = 3.71, *p* < .001) and slope (*M* = .21, *p* < .001) were both significant, indicating a significant decrease of avoidance from pre-treatment to 18-months follow-up.

**Supporting Information B**

We ran supplemental, post-hoc analyses examining the longitudinal links between attachment and youth mental health problems to elucidate the directional relation between these constructs. We used random-intercepts cross-lagged panel modeling (RI-CLPM) which allowed us to separate between-person variability from within-person variability. This allows us to examine how within-person changes in each variable relate to subsequent within-person changes in other variables. A conceptual diagram of the analysis is shown in Figure S1. Since the model was used to examine within-person changes, time invariant control variables were not added to the model.

**Parent-Reported Models**

The findings for parent-reported youth internalizing problems are shown in Figure S2. Attachment anxiety at the midpoint of the intervention (T2) predicted internalizing problems at post-intervention (T3). Internalizing problems did not predict either attachment variable longitudinally.

The findings for parent-reported youth externalizing problems are shown in Figure S3. Attachment avoidance at three timepoints from Post-intervention (T3) to 12-month follow-up (T5) predicted changes in externalizing problems at subsequent timepoints, from 6-month follow up (T4) until 18-month follow-up (T6). Externalizing problems did not predict either attachment variable longitudinally.

**Youth-Reported Models**

The findings for youth-reported youth internalizing problems are shown in Figure S4. Internalizing problems at pre-intervention (T1) predicted attachment avoidance at mid-intervention (T2). Neither attachment variable predicted internalizing longitudinally.

The findings for youth-reported youth externalizing problems are shown in Figure S5. Avoidant attachment at 6-month follow-up (T4) predicted externalizing problems at 12-month follow-up (T5). In turn, externalizing problems at 12-month follow-up predicted attachment anxiety at 18-month follow-up (T6).

**Figure S1**. Conceptual figure of the random intercepts cross-lagged panel model (RI-CLPM).


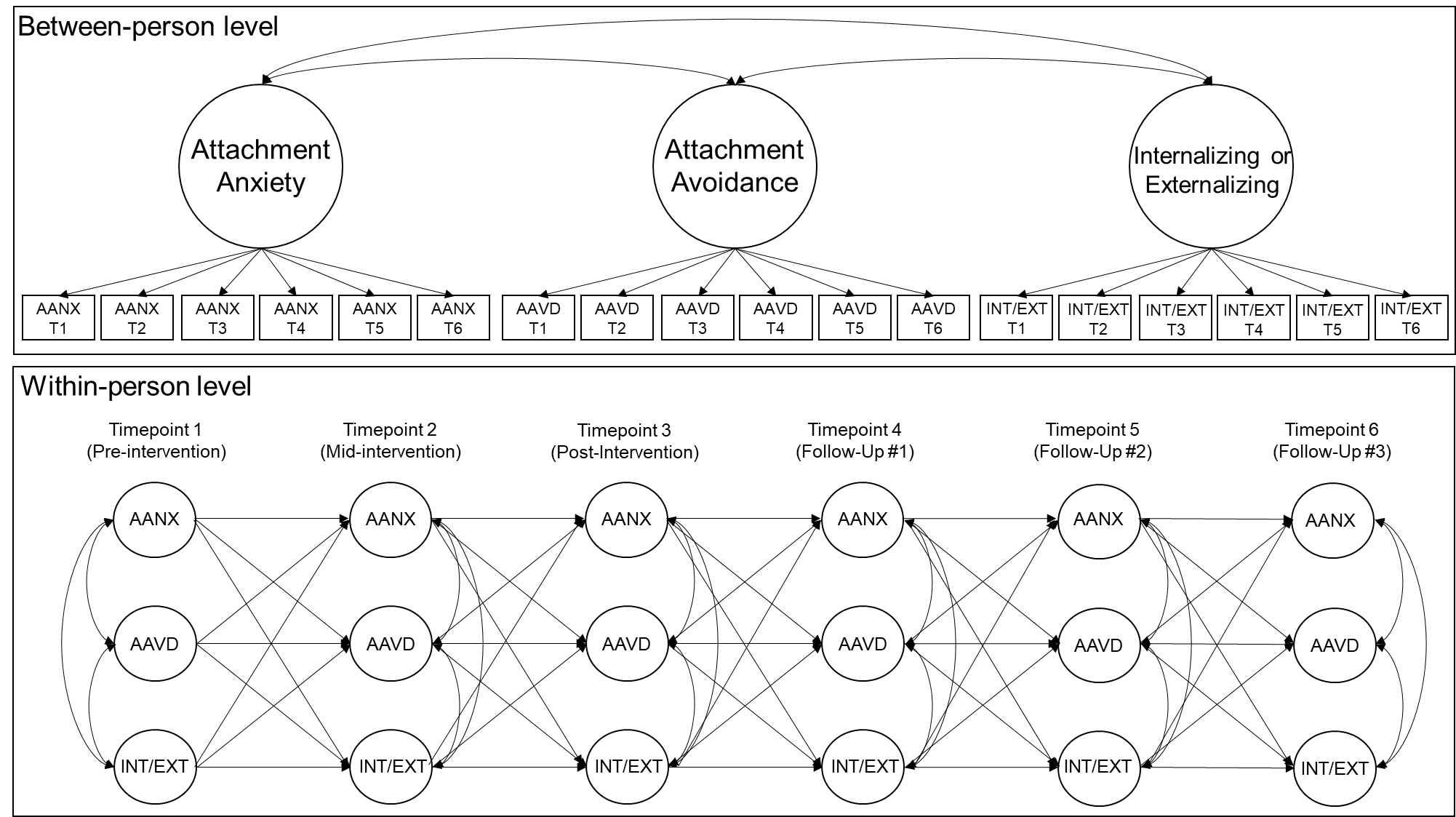


**Figure S2**. RI-CLPM for Parent Reports of Youth Internalizing Problems.
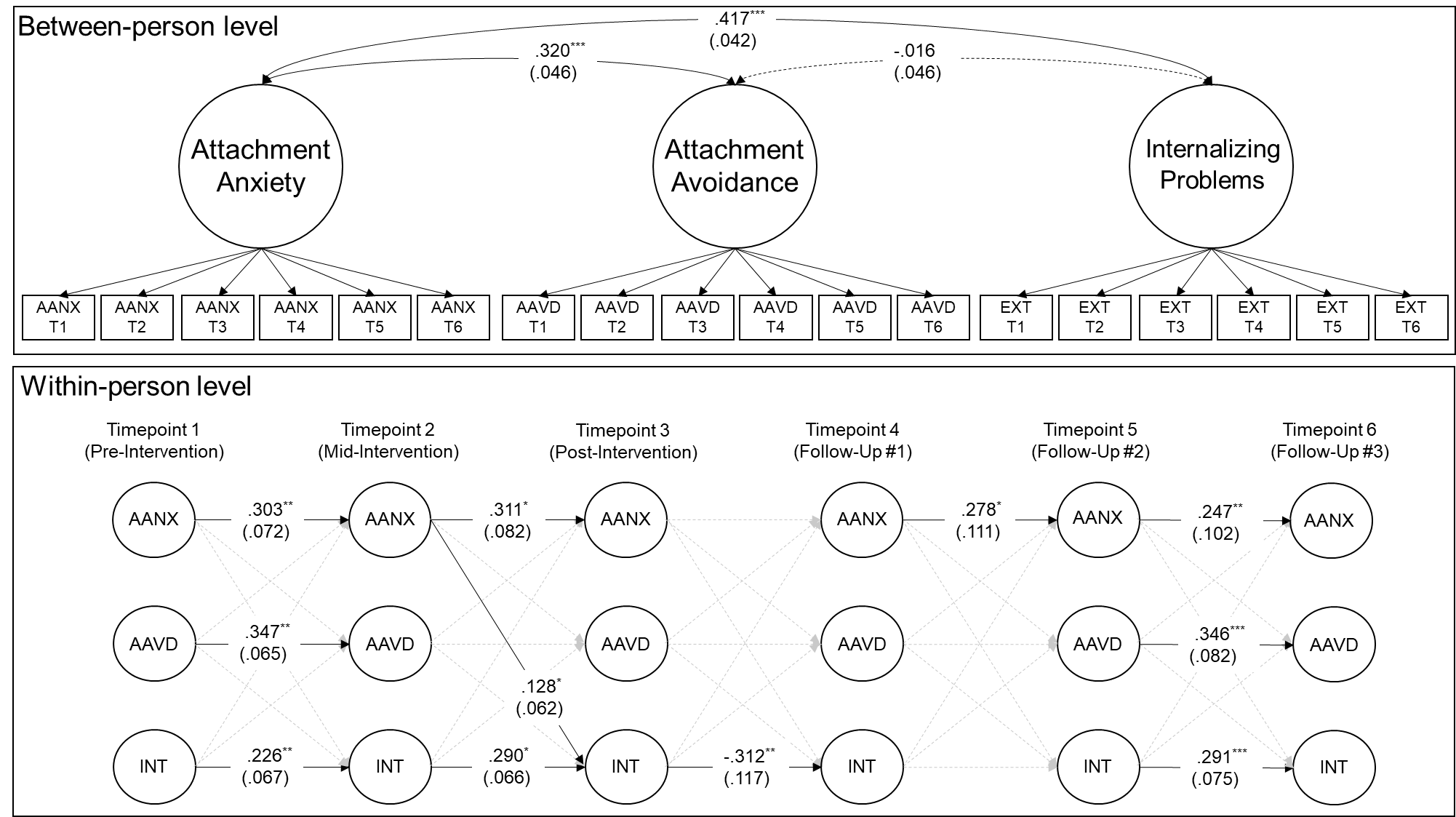


*Note*. AANX = Attachment anxiety; AAVD = Attachment avoidance; INT = Internalizing problems. Model fit was excellent: χ^2^(84) = 147.36, *p* < .001; RMSEA = .033, 90% CI [.024, .042]; CFI = .988; SRMR = .041. Parameter estimates represent standardized coefficients with standard errors in brackets. Only significant parameter estimates are shown. Greyed and broken lines represent non-significant paths. Within timepoint correlations are suppressed.

**Figure S3**. RI-CLPM for Parent Reports of Youth Externalizing Problems.


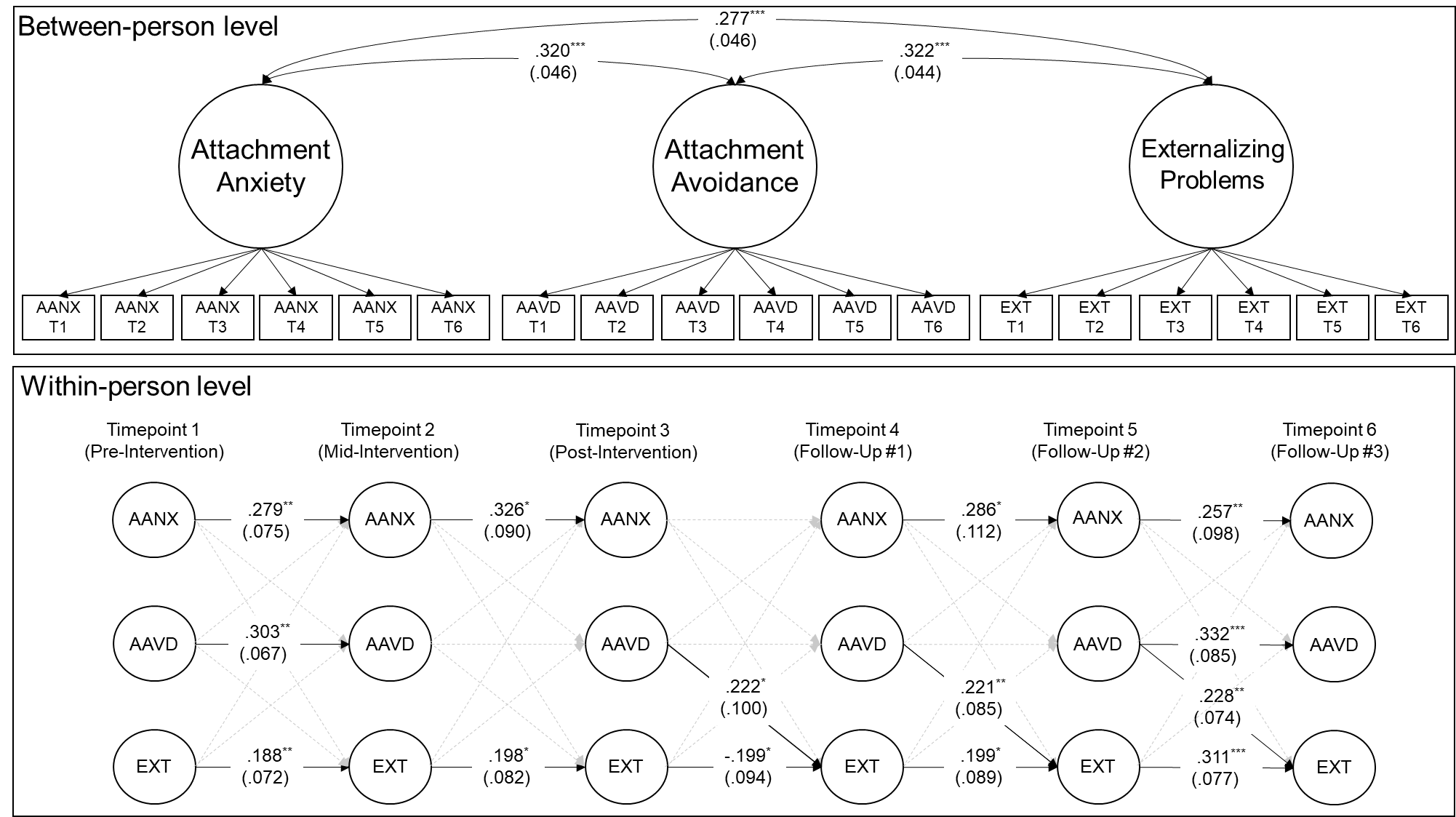


*Note*. AANX = Attachment anxiety; AAVD = Attachment avoidance; EXT = Externalizing problems. Model fit was excellent: χ^2^(84) = 132.05, *p* < .001; RMSEA = .029, 90% CI [.019, .038]; CFI = .990; SRMR = .045. Parameter estimates represent standardized coefficients with standard errors in brackets. Only significant parameter estimates are shown. Greyed and broken lines represent non-significant paths. Within timepoint correlations are suppressed.

**Figure S4**. RI-CLPM for Youth Reports of Youth Internalizing Problems.
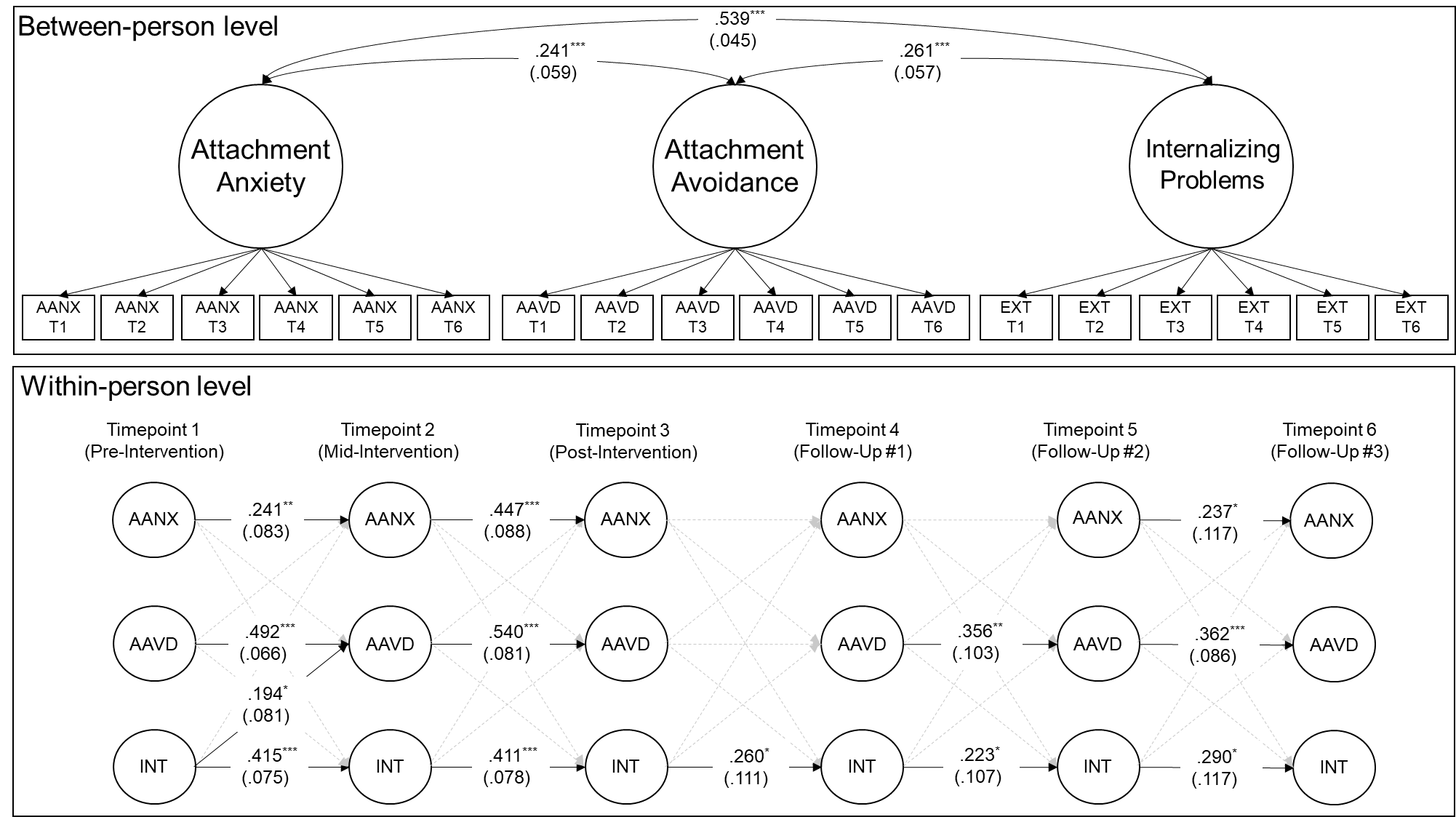
*Note*. AANX = Attachment anxiety; AAVD = Attachment avoidance; INT = Internalizing problems. Model fit was excellent: χ^2^(84) = 120.72, *p* = .005; RMSEA = .029, 90% CI [.016, .040]; CFI = .989; SRMR = .039. Parameter estimates represent standardized coefficients with standard errors in brackets. Only significant parameter estimates are shown. Greyed and broken lines represent non-significant paths. Within timepoint correlations are suppressed.

**Figure S5**. RI-CLPM for Youth Reports of Youth Externalizing Problems.


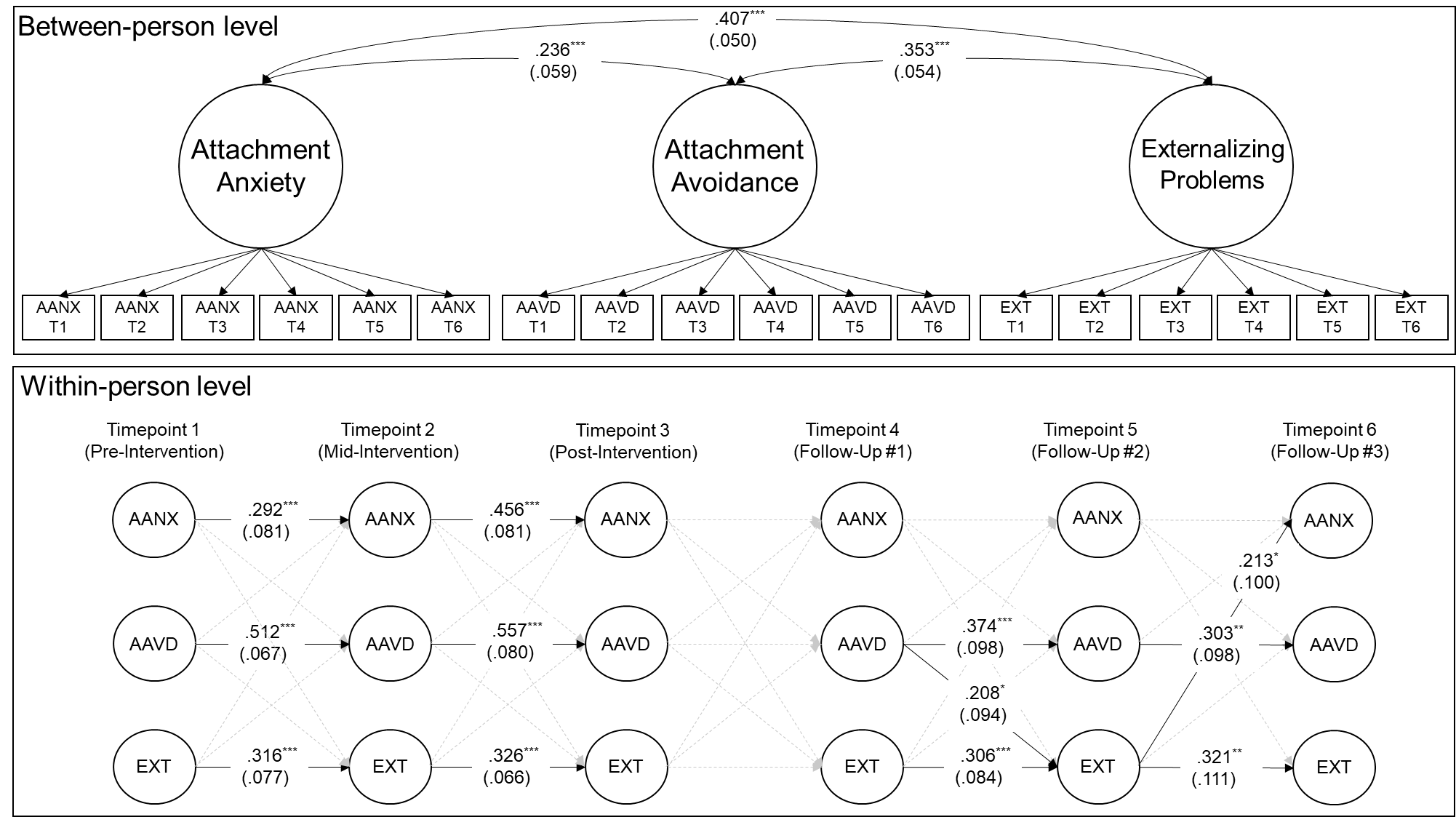
*Note*. AANX = Attachment anxiety; AAVD = Attachment avoidance; EXT = Externalizing problems. Model fit was excellent: χ^2^(84) = 111.62, *p* = .02; RMSEA = .025, 90% CI [.010, .037]; CFI = .992; SRMR = .038. Parameter estimates represent standardized coefficients with standard errors in brackets. Only significant parameter estimates are shown. Greyed and broken lines represent non-significant paths. Within timepoint correlations are suppressed.
